# Protective effect of homogeneous polysaccharides of Wuguchong (HPW) on intestinal mucositis induced by 5-fluorouracil in mice

**DOI:** 10.1186/s12986-022-00669-1

**Published:** 2022-05-18

**Authors:** Peng Shi, Tianqi Zhao, Wendong Wang, Fangli Peng, Ting Wang, Yong Jia, Linxuan Zou, Peng Wang, Simengge Yang, Yue Fan, Junwei Zong, Xueling Qu, Shouyu Wang

**Affiliations:** 1 Department of Orthopaedic Surgery, The First Affiliated Hospital of Dalian Medical University, 222 Zhongshan Road, Dalian, China; 2 College of Integrative Medicine, Dalian Medical University, 9 South Lushun Road West, Dalian, China; 3 Pelvic Floor Repair Centre, The Affiliated Dalian Maternity Hospital of Dalian Medical University, 1 Dunhuang Road, Dalian, China; 4 Pelvic Floor Repair Centre, Dalian Women and Children Medical Centre (Group), No. 1 Road of Sports New Town, Dalian, China; 5 Dalian Runxi Technology Development Co., Ltd, 3 Jinxia Street, Dalian, China; 6 Department of Orthopaedics, The Second People’s Hospital of Dalian, 29 Hongji Street, Dalian, China

**Keywords:** Drug side effects, Intestinal mucositis, Polysaccharide, Wuguchong, Intestinal barrier, 5-Fluorouracil

## Abstract

**Background:**

In hospitalized patients, drug side effects usually trigger intestinal mucositis (IM), which in turn damages intestinal absorption and reduces the efficacy of treatment. It has been discovered that natural polysaccharides can relieve IM. In this study, we extracted and purified homogenous polysaccharides of Wuguchong (HPW), a traditional Chinese medicine, and explored the protective effect of HPW on 5-fluorouracil (5-FU)-induced IM.

**Methods and results:**

First, we identified the physical and chemical properties of the extracted homogeneous polysaccharides. The molecular weight of HPW was 616 kDa, and it was composed of 14 monosaccharides. Then, a model of small IM induced by 5-FU (50 mg/kg) was established in mice to explore the effect and mechanism of HPW. The results showed that HPW effectively increased histological indicators such as villus height, crypt depth and goblet cell count. Moreover, HPW relieved intestinal barrier indicators such as D-Lac and diamine oxidase (DAO). Subsequently, western blotting was used to measure the expression of Claudin-1, Occludin, proliferating cell nuclear antigen, and inflammatory proteins such as NF-κB (P65), tumour necrosis factor-α (TNF-α), and COX-2. The results also indicated that HPW could reduce inflammation and protect the barrier at the molecular level. Finally, we investigated the influence of HPW on the levels of short-chain fatty acids, a metabolite of intestinal flora, in the faeces of mice.

**Conclusions:**

HPW, which is a bioactive polysaccharide derived from insects, has protective effects on the intestinal mucosa, can relieve intestinal inflammation caused by drug side effects, and deserves further development and research.

## Background

Approximately 30% of in-hospital patients are at risk of malnutrition [1], which is mostly caused by reduced food intake, malabsorption, loss of gastrointestinal nutrients or increased energy expenditure. One important reason for this risk is malabsorption, which is associated with the loss of epithelial integrity and impaired epithelial transport in the gastrointestinal tract [2]. This pathological process not only occurs during disease development but can also be caused by drug side effects. For example, according to research statistics, 40% of cancer patients receiving chemotherapy develop lower gastrointestinal mucositis [3], which leads to malnutrition.

5-fluorouracil(5-FU)is an anti-metabolic anticancer drug that is widely used in the treatment of cancer. However, a large proportion of patients using 5-FU develop intestinal mucositis (IM) [4]. Experimental studies have shown that 5-FU can decrease crypt and villi length by triggering apoptosis in intestinal epithelial cells [5]. In addition, after 2 days of 5-FU treatment, the nuclear factor kappa-B (NF-κB)was highly overactive in the small intestine. Various inflammatory mediators, including tumour necrosis factor-α (TNF-α), are involved in the process of IM [6]. Furthermore, among the molecular events that cause intestinal mucosal inflammation, chemotherapeutic drugs lead to an imbalance of intestinal flora, which in turn leads to intestinal mechanical barrier and mucosal barrier dysfunction [7].

The treatment of small IM remains difficult, and the traditional zinc derivatives, loperamide or mesalazine do not have the desired effectiveness [8]. Therefore, searching for natural products that can treat IM is essential to protect against gastrointestinal damage and reduce inflammation. It has been shown that natural dietary polysaccharides can cure IM by promoting the development of epithelial cells and mucosal immune cells, enhancing intestinal barrier function, and thus promoting nutrient absorption.

For example, homogenous polysaccharides extracted from Dendrobium huoshanense have regulatory effects on both intestinal and systemic immunity, and it can improve IM by improving mucosal barrier function and microbial composition in different regions of the intestine [9]. Sea cucumber fucoidan (SC-FUC) improves intestinal tissue architecture, including indicators such as villi height and crypt depth, and ameliorates immune imbalance by regulating the Th1/Th2 ratio to counteract small intestinal mucosal damage [10]. However, the biological activity of insect polysaccharides has not been properly explored compared with those of many natural organisms, such as plants, fungi and marine organisms. Previously, glycosaminoglycan from dung beetles exhibited anticancer properties, and Huechys sanguinea glycosaminoglycan has been used to treat tuberculous amenorrhea and scabies [11]. A novel polysaccharide extracted from the larvae of the black soldier fly (BSF) (*Hermetia illucens*) acts as an immune activator by stimulating RAW264.7 cells through the TLR signalling pathway [12].

The traditional Chinese medicine Wuguchong is a kind of natural medicine produced by maggots; it is the dried larva of *Chrysomya megacephala* or other related insects of Calliphoridae. Ancient Chinese medicine books recorded that its flavour is salty and sweet and that its nature is cold. Using this medicine invigorates the spleen, eliminates food accumulation, clears heat and eliminates infantile malnutrition. Previous studies have shown that polysaccharides extracted from Wuguchong (PEW) could be used as bioactive agents to prevent obesity [13]. Moreover, in that study, researchers tentatively explored the role of PEW in regulating intestinal microbial composition and maintaining intestinal epithelial integrity, which can reduce the ratio of Firmicutes to Bacteroides and the relative abundance of Proteobacteria in high-fat fed mice and improve the expression of tight junction proteins. On the other hand, the fatty acid extract of Wuguchong has been shown to promote wound healing on the surface of the body and promote the proliferation and migration of endothelial cells [14].

In this study, the polysaccharides were further purified, homogeneous polysaccharides of Wuguchong (HPW) was obtained by gel column chromatography, and its protective effect on IM induced by 5-FU was studied in mice. HPW protects the mechanical and immune barriers of the small intestine by improving the morphology of small intestinal villi and promoting goblet cell proliferation and tight junction protein expression. In addition, in the current study, we assessed the amount of short-chain fatty acids (SCFAs) in the faeces of mice. Based on previous studies [13], we hypothesized that HPW may protect against chemotherapeutic drug-induced intestinal mucosal damage through the intestinal flora and metabolic pathways to promote nutrient absorption.

## Methods

### Extraction of HPW

The crude extract of polysaccharides from Wuguchong was prepared by water extraction and alcohol precipitation according to our previous method [13]. Briefly, dried insect powder was boiled three times in 95% alcohol in a Soxhlet reflux machine to remove excess lipids. A solid–liquid ratio of 20 mg/L was kept slightly boiling at 110 °C, aqueous extraction for 6 h. The concentrated solution was precipitated with 3 times ethanol for 2–3 days, the supernatant was poured, frozen and dried into powder form, and the crude polysaccharide extract was obtained.

**Fig. 1:**
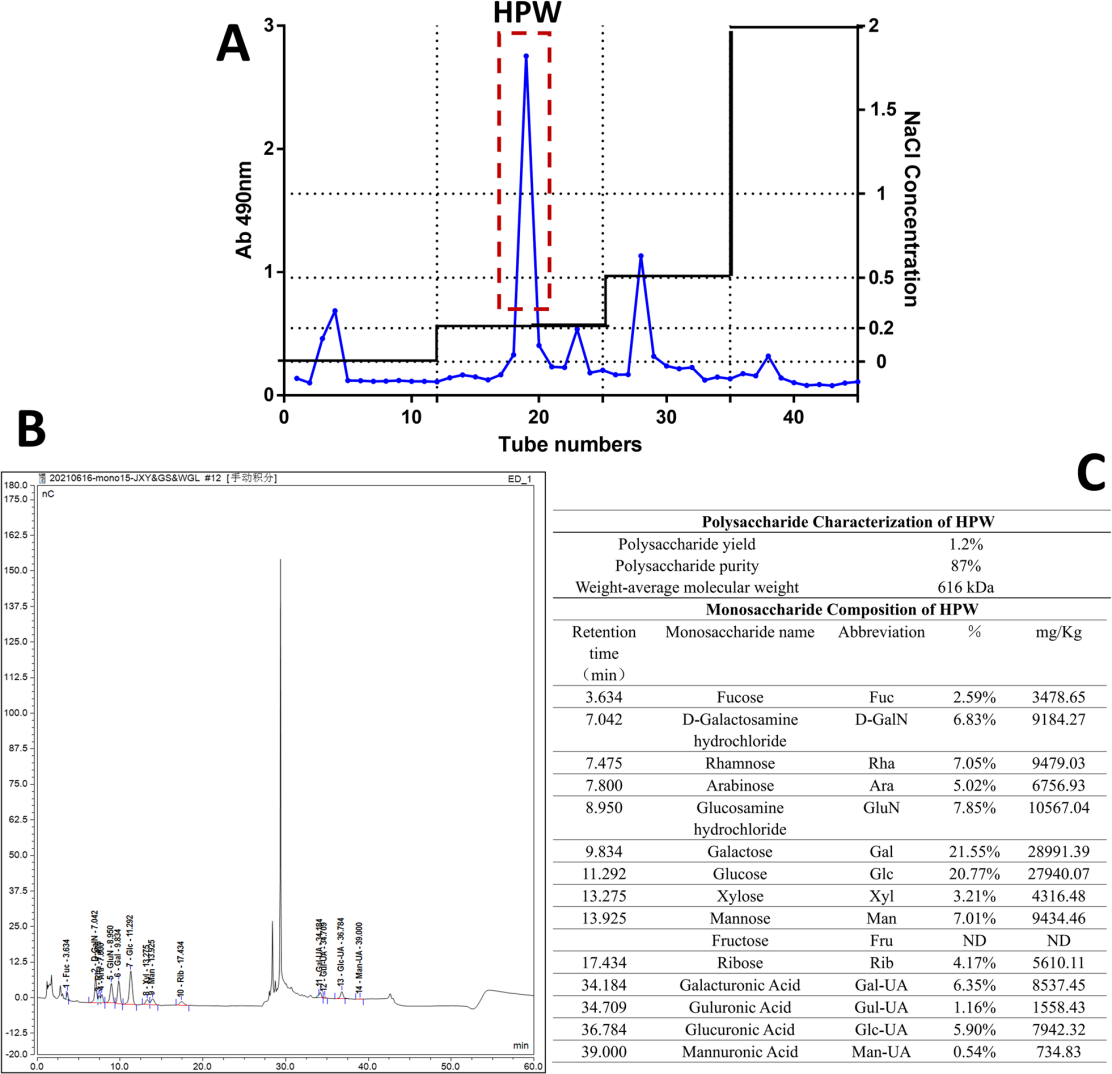
Purification and identification of HPW. **A** Elution curves of crude polysaccharides and the position of HPW. The abscissa is the number of elution tubes, and the ordinate is the absorbance value of the tracking sugar levels at 490 nm by the phenol–sulfuric acid method. **B** Ion chromatogram of the monosaccharide composition of HPW samples. The abscissa is the retention time (Time, min), and the ordinate is the response value of the ion detection (Response, nC). **C** Aggregated table of properties and monosaccharide composition of HPW

After protein removal by the Sevage method, the crude extract was separated and purified by the DEAE-cellulose column chromatography method to obtain homogenous polysaccharides with similar molecular weights and the same polarity. DEAE Sepharose Fast Flow packing was eluted with distilled water to a neutral pH condition, the flow rate was adjusted to 5 mL/min, and this procedure was maintained for 2 h. The crude polysaccharides were dissolved in distilled water, followed by stepwise elution with distilled water and 0.2, 0.5 and 2.0 M NaCl solutions at a flow rate of 15 mL/min. The phenol–sulfuric acid method was used for tracking and detection. A microplate tester was used for detection at 490 nm, and a scatter diagram was drawn (Fig. 1A).

According to the peak shape, each component was collected, concentrated, dialysed in a 3.5 kDa molecular weight cut-off membrane, and freeze-dried. The fractions that eluted with 0.2 M NaCl were further purified on a Sephacryl S-200 column (1.6 × 80 cm) and were eluted with potassium phosphate buffer (PBS, 0.1 M, pH 7.2) at a flow rate of 0.5 mL/min [15].

Finally, a component with a relatively high concentration was obtained, which was named HPW. The extraction rate was 1.2%, and the purity was 87% by the phenol–sulfuric acid method.

### Characterization and monosaccharide composition of HPW

The molecular weight and purity of HPW were assayed with high-performance gel permeation chromatography (HPGPC) (Shimadzu, Japan) using dextrans (MW: 5 kDa, 12 kDa, 25 kDa, 50 kDa, 80 kDa, 150 kDa, 270 kDa, Sigma–Aldrich, USA) as standard samples in a TSK-gel GMPWXL (7.8 mm × 300 mm, 5 μm) [16]. The data showed that the average molecular weight of HPW was approximately 616 kDa, and the purity was 87% (Fig. 1C).

High-performance anion-exchange chromatography (HPAEC) was used to identify the monosaccharide composition of HPW. The chromatographic system used a Thermo ICS5000 ion chromatography system (Thermo Fisher Scientific, USA), and an electrochemical detector was used to analyse the monosaccharide components with the following parameters: flow rate, 0.5 mL/min; injection volume, 5 μL; solvent system, 0.1 M NaOH: (0.1 M NaOH, 0.2 M NaAc); gradient program, 95:5 V/V at 0 min, 80:20 V/V at 30 min, 60:40 V/V at 30.1 min, 60:40 V/V at 45 min, 95:5 V/V at 45.1 min, 95:5 V/V at 60 min.

The data showed that HPW was mainly composed of galactose (21.55%), glucose (20.77%), rhamnose (7.05%), mannose (7%), arabinose (5.02%) and xylose (3.21%). An ion chromatogram of the samples is shown in Fig. 1B, and the monosaccharide composition is detailed in Fig. 1C.

### Animals

SPF-grade C57BL/6 male mice weighing 18–22 g and aged 6–8 weeks were purchased from the Experimental Animal Centre of Dalian Medical University, China. All mice were adaptively fed for 7 days for subsequent experiments. The ambient temperature was 20–25 °C, and the relative humidity was 40–60%. Mice were maintained on a 12-h light–dark cycle and were randomly given chow and drinking water. The procedures for animal experiments were performed strictly in accordance with the standard guidelines for laboratory animals and approved by the Ethics Committee of Dalian Medical University (Ethical Approval Number: AEE19074).

### Experimental procedure

Forty C57BL/6 male mice were randomly allocated into 5 groups with 8 mice in each group. The groupings were as follows:

- Group 1: Water + saline.
- Group 2: HPW (100 mg/kg BW) + saline.
- Group 3: Water + 5-FU (50 mg/kg BW).
- Group 4: HPW (100 mg/kg BW) + 5-FU (50 mg/kg BW).
- Group 5: Mesalazine (10 mg/kg BW) + 5-FU (50 mg/kg BW).

To obtain a stable experimental animal model of IM, we used a previously reported protocol [17]: normal saline and 5-FU (50 mg/kg) were injected intraperitoneally during the first three days. Water, HPW or mesalazine was administered orally 1 h before 5-FU administration for one week.

### Physical manifestations and tissue collection

The body weight and diarrhoea score of each mouse and the food intake of each group were recorded every day during the experiment. Diarrhoea severity was scored daily by an uninformed researcher based on criteria in previous studies [18]. The scoring criteria for diarrhoea severity were as follows: 0: normal stool; 1: slight (wettish and soft stool); 2: moderate (unformed stool, wet crissum and stained coat); and 3: severe (watery stool).

At the end of the experiment (7 days after treatment), fresh faeces were collected and quickly placed into liquid nitrogen for preservation and used for SCFA analysis. Mice were deprived of water and food for 12 h. Blood was collected after pentobarbital anaesthesia for enzyme-linked immunosorbent assay (ELISA) analysis. The entire small intestine was rapidly dissected. A 2-cm intestinal segment of the jejunum was taken 15 cm behind the pylorus and fixed with 4% paraformaldehyde for haematoxylin–eosin (HE) staining and periodic acid-Schiff (PAS) staining. The rest of the small intestine was used for other molecular biological experiments.

### Histological analysis

The 2-cm jejunum segment was divided into two portions and placed in 4% paraformaldehyde fixative and Karnovsky fixative. After paraformaldehyde fixation for 12–24 h, the tissues were subjected to procedures such as dehydration and paraffin embedding. Slices with a thickness of 5 μm were cut and rehydrated with graded ethanol after being dewaxed with xylene. HE staining was then performed, the dye was washed off with water, and the slices were dehydrated and sealed. Villus length and crypt depth, which are specific indicators of intestinal barrier function and absorption, were measured under a microscope (Olympus BX-40, Japan) using Image-Pro Plus 6.0 software.

The other part of the jejunal tissue was fixed in Karnovsky buffer and then similarly processed according to dehydration, embedding and sectioning procedures. The sections were stained with Schiff's reagent for 20 min, followed by haematoxylin for 20 min. The staining solution was washed away, and the slices were subsequently dehydrated and sealed. The number of goblet cells on each villus was counted by Image-Pro Plus 6.0 software. Goblet cells appear purplish red under a microscope and are an important indicator of the small intestinal mucosal barrier. Reagents were provided by Wuhan Servicebio Biotechnology Co., Ltd.

### Reagents and antibodies

A Diamine oxidase activity (DAO) detection kit was purchased from Beijing Solarbio Technology Co., Ltd. A total antioxidant capacity (T-AOC) test kit was obtained from Nanjing Jiancheng Biological Engineering Research Institute Co., Ltd. ELISA kits (Shanghai Langton Biotechnology Co., Ltd.)were used to measure D-lactate, a mechanical barrier marker, and SIgA, a mucosal barrier marker. Claudin-1, proliferating cell nuclear antigen (PCNA), TNF-α and GAPDH antibodies were provided by Wuhan Proteintech Biotechnology Co., Ltd. Occludin, NF-κB (P65) and COX-2 antibodies were purchased from Abcam (Cambridge Science Park in Cambridge, UK). Mouse IL-10 and IL-1β ELISA kits was purchased from Beijing Solarbio Technology Co., Ltd.

### Western blot analysis

The expression of Claudin-1, Occludin, PCNA, TNF-α, NF-κB (P65) and COX-2 in jejunum and ileum tissue samples was measured by Western blotting. Tissue samples were lysed with RIPA buffer containing protease and phosphatase inhibitors to obtain total proteins, followed by BCA protein quantification. Related reagents were purchased from Sevenbio (Beijing) Co., Ltd. The lysed supernatant (approximately 80 μg of protein) was transferred to a PVDF membrane (Millipore, USA) by 10% SDS–PAGE. After being blocked for two hours at room temperature in 5% nonfat milk, the membranes were incubated with the following primary antibodies overnight at 4 °C: Claudin-1 (1:1000), Occludin (1:2000), PCNA (1:5000), TNF-α (1:1000), NF-κB (p65) (1:1000), COX-2 (1:1000), and GAPDH (1:2000). RP-conjugated rabbit anti-mouse IgG and goat anti-rabbit IgG secondary antibodies were added and incubated at room temperature for 1 h, and the protein bands were visualized with an ECL detection system (Syngene, UK). Image-Pro Plus 6.0 software was used to analyse the grey values.

### Measurement of SCFAs

The SCFA levels in mouse faecal samples were measured according to a previously reported method [19]. An appropriate amount of the sample was added to 0.3 mL of water, 100 μL of 50% sulfuric acid, 25 μL of 500 mg/L internal standard (cyclohexanone) solution and 0.5 mL of ether, after which the mixed solution were homogenated for 1 min and centrifuged at 12,000 rpm at 4 °C for 10 min. The supernatant was placed on the instrument for testing (Gas Chromatograph-Mass Spectrometer, Shimadzu GCMS QP2010-Ultra, Japan). The chromatographic system was as follows: Agilent DB-WAX capillary column (30 m × 0.25 mm × 0.25 μm). The carrier gas was high purity helium (≥ 99.9%), and the flow rate was 1.0 mL/min. The inlet temperature was 220 °C, the injection volume was 1 μL, and the solvent delay time was 2.5 min for splitless injection. For the mass spectrometry system, an electron bombardment ion source (EI) was used, the ion source temperature was 230 °C, and the interface temperature was 220 °C. The chromatograph was connected to a microcomputer with a detector for collecting the results of the chromatographic analysis with the GC Solution program (Shimadzu, Japan).

### Statistical analysis

The data are presented as the means ± SEM and were analysed using one-way ANOVA with GraphPad Prism 8.0 followed by Dunnett’s test. We considered the data significant when *p* < 0.05.

## Results

### Effect of HPW on the physiological manifestations of mice

**Fig. 2:**
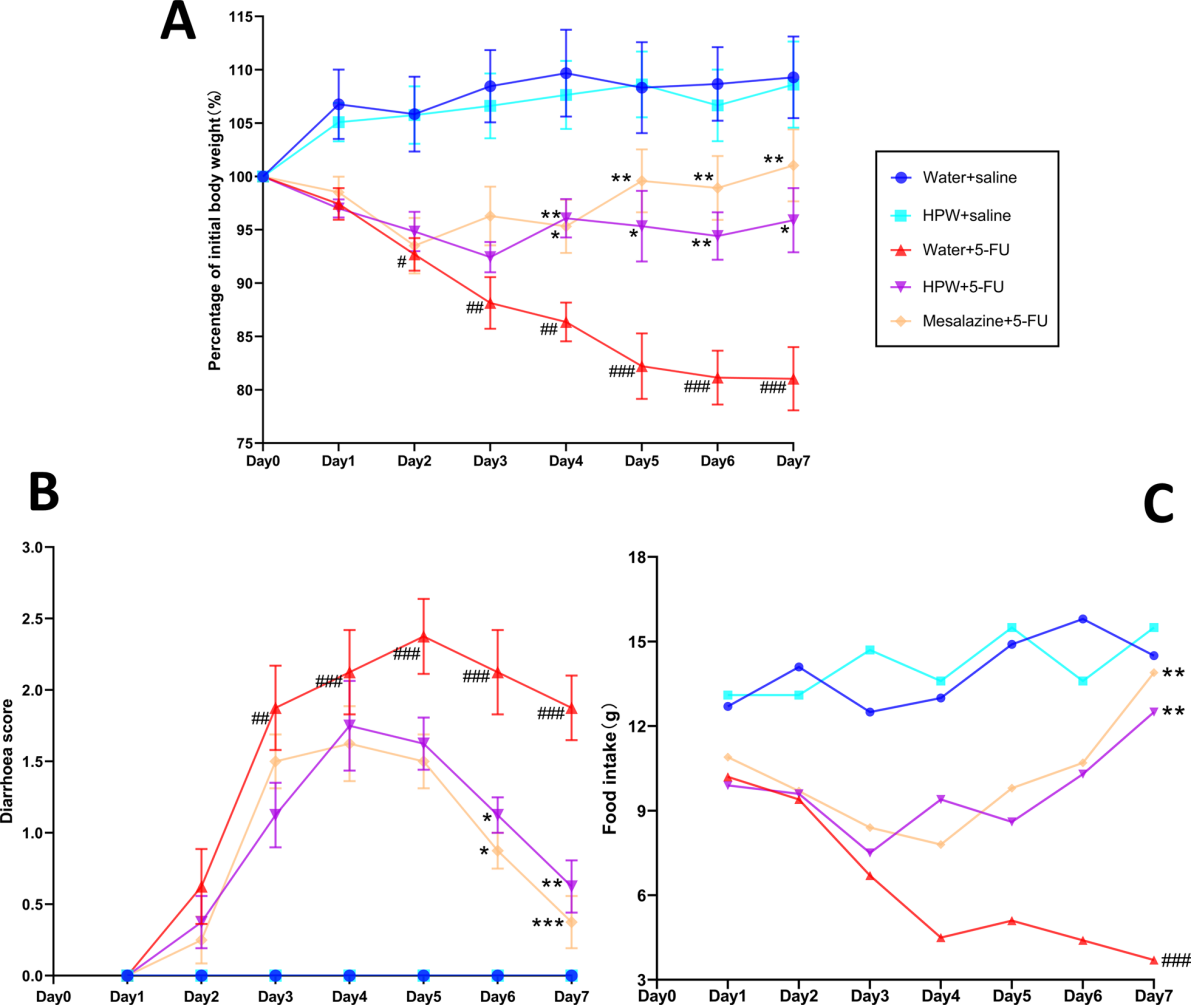
Effect of HPW on the physiological manifestations of mice. **A** Body weight change analysis. (Weight/initial weight) × 100%. **B** The diarrhoea scores of the mice. **C** Food intake of mice in each group. The data are presented as the means ± SEM and were analysed using one-way ANOVA followed by Dunnett’s test (n = 8). “*” represents the comparison with the model group (water + 5-FU), and “#” represents the comparison with the control group (water + saline). One tag means *p* < 0.05, two tokens represent *p* < 0.01, and three indicates *p* < 0.001

Body weight changes, the diarrhoea index, and food intake are important phenotypic indicators of intestinal mucosal inflammation. In the present study, no significant weight loss, decline in food intake, diarrhoea, or death was observed in the control group (water + saline). Compared with the control group, the modelling group (water + 5-FU) experienced significant weight loss from days 2 and 3, accompanied by diarrhoea (unformed or even watery stools) and reduced food intake (Fig. 2). However, after HPW administration, the conditions improved. From the fourth day onwards, weight loss (Fig. 2A) and diarrhoea scores (Fig. 2B) were relieved in the HPW + 5-FU group, and food intake gradually resumed (Fig. 2C). Therefore, we hypothesized that HPW protects against IM. We also evaluated the physiological status of mice that were intragastrically administered HPW alone. As shown in Fig. 2, there was no significant difference between the HPW + saline group (the light blue curve) and the control group, indicating that supplementation with HPW alone did not have adverse effects. In this part of the experiment, we used mesalazine as a positive control, and the physical status of the mice in the mesalazine + 5-FU group improved, as anticipated.

### Effect of HPW on the histological status of IM in mice.

**Fig. 3:**
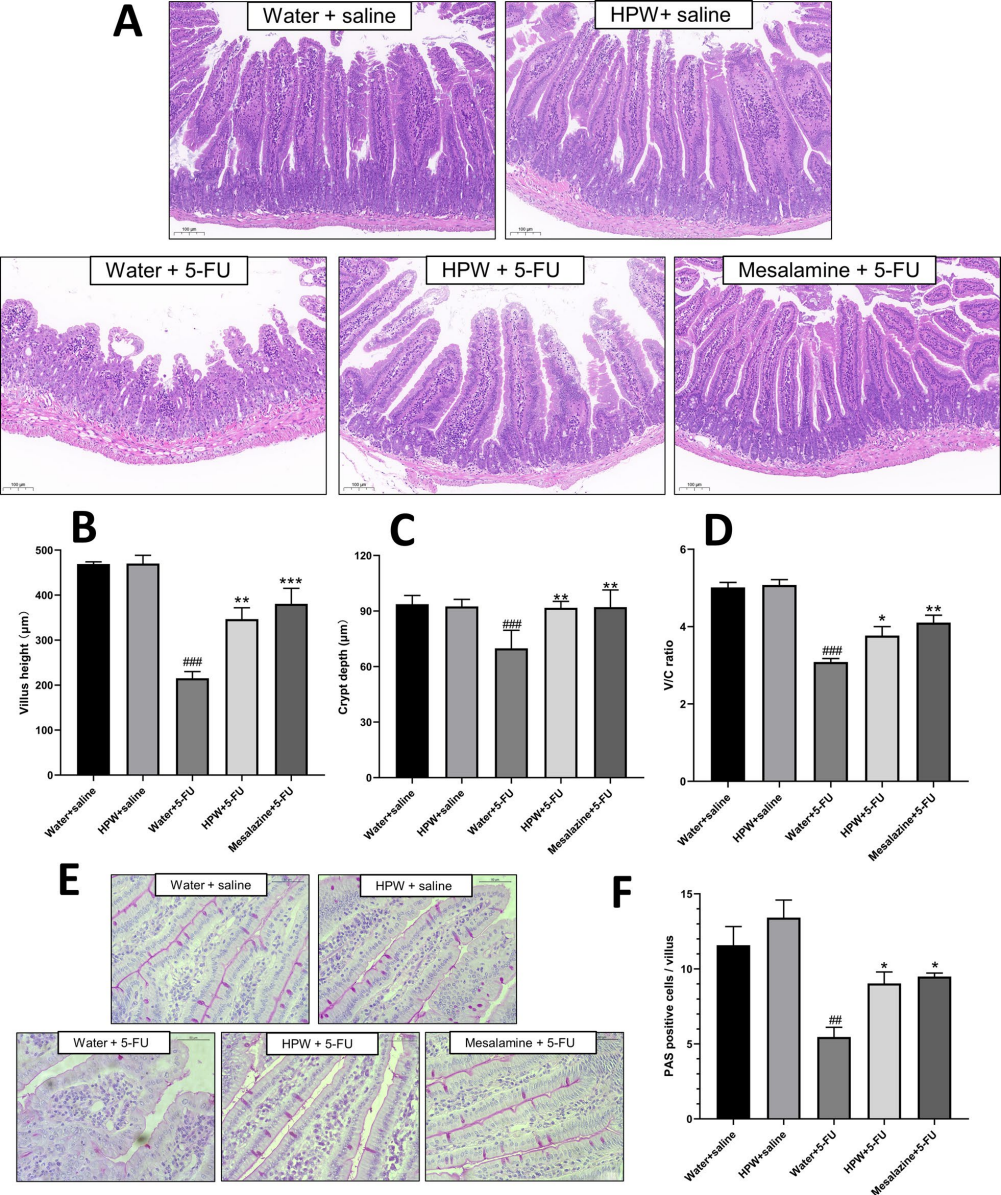
Microhistological examination of the small intestine. **A** HE staining of intestine tissues (scale bar = 100 μm). **B** Villus height. **C** Crypt depth. **D** The ratio of V/C. **E** PAS staining of goblet cells (scale bar = 50 μm). **F** Analysis of goblet cell counts. The data are presented as the means ± SEM and were analysed using one-way ANOVA followed by Dunnett’s test (n = 8). “*” represents the comparison with the model group (water + 5-FU), and “#” represents the comparison with the control group (water + saline). One tag means *p* < 0.05, two tokens represent *p* < 0.01, and three is *p* < 0.001

Figure 3A shows that the cross sections of the jejunum in the fluorouracil-treated group (water + 5-FU) exhibited severely damaged pathological structures, showing significantly decreased villus height, decreased crypt depth and morphological dysplasia. Vacuolar oedema and inflammatory cell infiltration were observed in the submucosa and muscularis. HPW and mesalazine treatment alleviated the histopathological damage to different degrees. The intestinal villus height, crypt depth, and villus crypt ratios were markedly enhanced in the HPW + 5-FU group (Fig. 3B–D), demonstrating a protective effect against chemotherapeutic drug-induced mucosal injury. Compared with the response in the control group, intragastric administration of HPW alone had no effect on the small intestinal micromorphology.

### Analysis of goblet cell counts and sIgA secretion

**Fig. 4:**
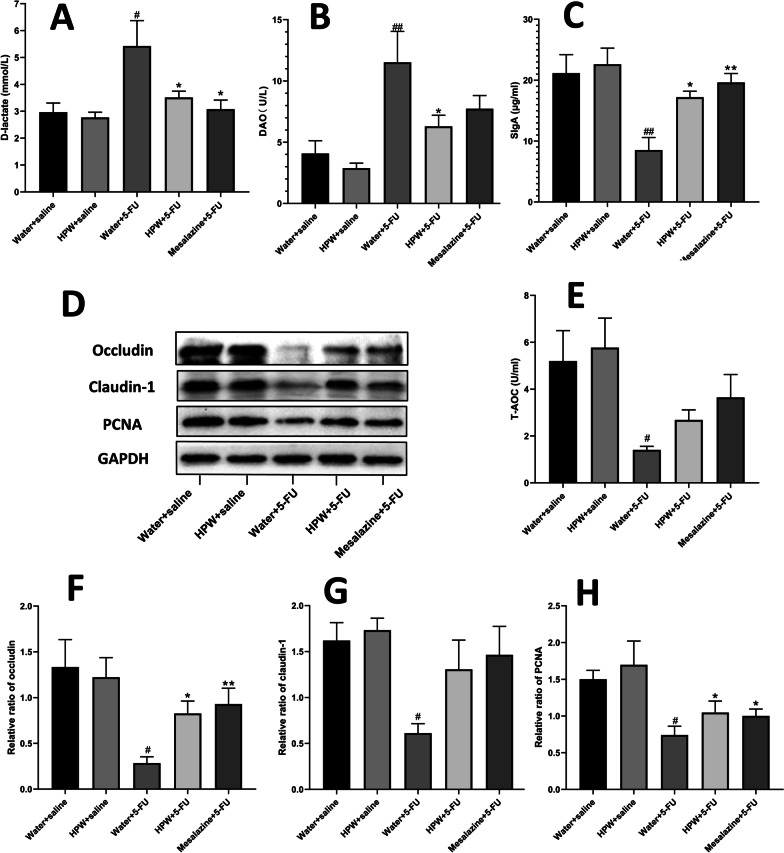
Effect of HPW on small intestinal barrier indices. **A** D-lactic acid (D-LA), **B** DAO and **E** total antioxidant capacity (T-AOC) in serum. **C** SIgA in tissue homogenate. **D** Contrast diagram showing Occludin, Claudin-1, PCNA and GAPDH protein bands. **F**–**H** Analysis of their respective greyscale values. The data are presented as the means ± SEM and were analysed using one-way ANOVA followed by Dunnett’s test (n = 8). The “*” represents the comparison with the model group (water + 5-FU), and “#” represents the comparison with the control group (water + saline). One tag means *p* < 0.05, two tokens represent *p* < 0.01, and three are *p* < 0.001

Compared with the effect on the water + saline group, intraperitoneal injection of 5-FU markedly lowered goblet cell counts on each small intestinal villus (Fig. 3E). After with the administration of HPW and mesalazine, the cupped cell counts returned, and alignment returned to normal with statistically significant differences. There was no statistically significant difference between the control group and the HPW alone group (Fig. 3F). The same trend was also observed in the levels of intestinal secreted IgA, as detected by ELISA; that is, fluorouracil reduced the secretion of sIgA, and mesalazine and HPW significantly reversed these effects (Fig. 4C).

### Effects of HPW on the inflammation-associated proteins NF-κB, COX-2, TNF-α and the inflammtory cytokines IL-1β, IL-10

**Fig. 5:**
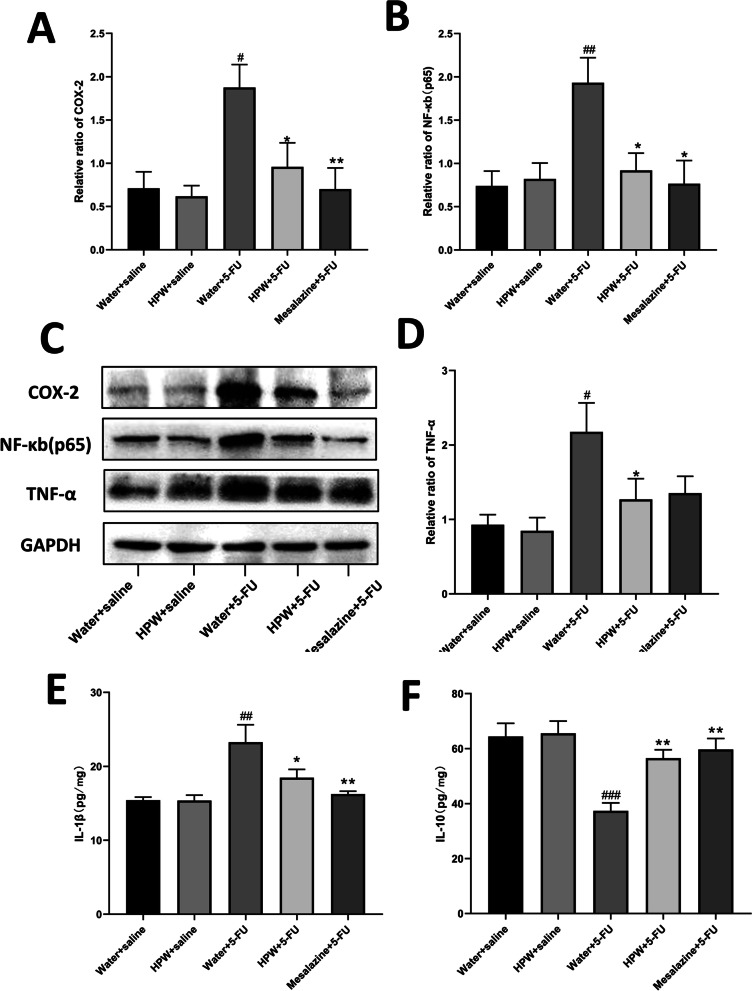
Effect of HPW on the inflammatory pathway and inflammatory factors. Greyscale value analysis of **A** COX-2, **B** NF-κB and **D** TNF-α. **C** Contrast diagram showing these three molecules and GAPDH protein bands. Analysis of **E** IL-1β, **F** IL-10 in tissue homogenate. The data are presented as the means ± SEM and were analysed using one-way ANOVA followed by Dunnett’s test (n = 8). “*” represents the comparison with the model group (water + 5-FU), and “#” represents the comparison with the control group (water + saline). One tag means *p* < 0.05, two tokens represent *p* < 0.01, and three are *p* < 0.001

The effects of HPW on the intestinal expression levels of NF-κB, COX-2 and TNF-α are shown in Fig. 5. Compared with those in the control group, the expression levels of these three key targets in the water + 5-FU group were significantly enhanced. This finding suggests activation of inflammatory pathways at the molecular level. HPW reversed the negative effects of 5-FU (Fig. 5A–D). Activation of NF-κB promotes the secretion of inflammtory cytokines. Figure 5E, F showed that HPW could not only considerably decrease the levels of IL-1β but also increase IL-10 expression in IM mice.

### Analysis of SCFAs

**Fig. 6:**
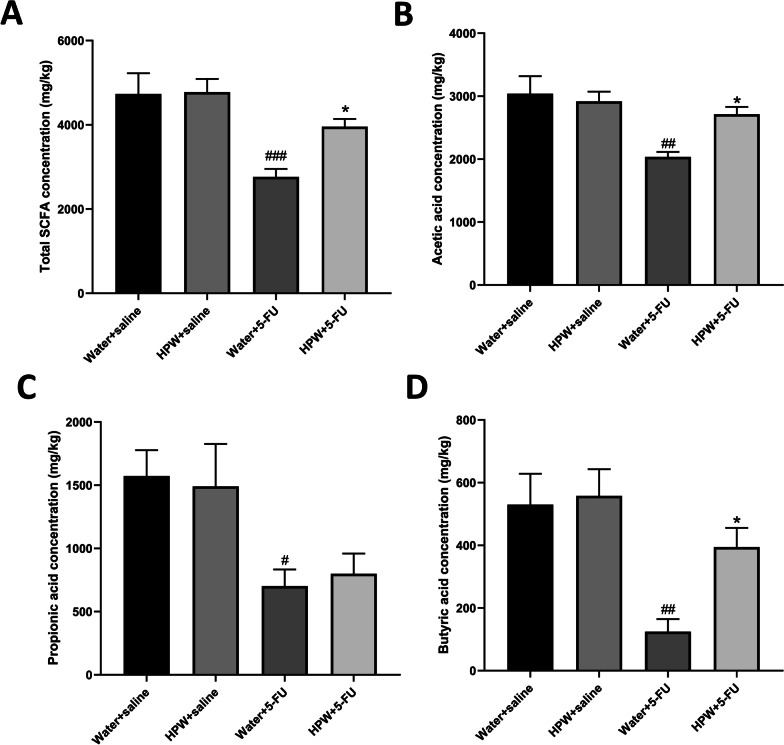
Effect of HPW on SCFAs. Analysis of **A** total SCFAs, **B** acetic acid, **C** propionic acid, and **D** butyric acid. The data are presented as the means ± SEM and were analysed using one-way ANOVA followed by Dunnett’s test (n = 8). “*” represents the comparison with the model group (water + 5-FU), and “#” represents the comparison with the control group (water + saline). One tag means *p* < 0.05, two tokens represent *p* < 0.01, and three are *p* < 0.001

Figure 6A shows the changes in total SCFAs: the levels of faecal SCFAs in mice decreased after 5-FU treatment and increased after HPW supplementation. The same trend occurred in the acetic acid levels and was significant (Fig. 6B). Consistent with the previous two indices, the addition of 5-FU reduced the levels of propionic acid and butyric acid (Fig. 6C, D). However, the level of propionic acid (Fig. 6C) did not change after HPW gavage, while the level of butyric acid (Fig. 6D) was statistically rised. There was also no significant variation in mice administered HPW alone compared to control mice.

## Discussion

It has been extensively demonstrated that the biological activity of polysaccharides is related to their chemical characteristics, monosaccharide composition and the binding structure of glycosidic bonds [20]. Oral natural nonstarch polysaccharides can be rapidly degraded into low-molecular-weight polysaccharide fragments in the gastrointestinal tract, which are stably retained in the fluid of the stomach and small intestine to participate in the digestion and absorption process and are absorbed into the blood to participate in systemic circulation [21]. Our data show that there is 20.77% glucose in HPW, which is similar to many polysaccharides that exhibit intestinal mucosal protection. Glucose can be absorbed into the blood through internalization by intestinal epithelial cells, exerting anti-inflammatory and immunoregulatory effects [22].

The extent of intestinal injury induced by 5-FU was reported to be dose dependent, with moderate weight loss and diarrhoea in mice that were intraperitoneally injected with 50 and 100 mg/kg 5-FU. At this dose range, mouse mortality was low, and activity was stable [23]. In our experiment, typical pathogenic changes of IM in mice were observed after intraperitoneal injection of 5-FU (50 mg/kg) for three consecutive days (Day 1-Day 3), which was consistent with previous studies [17]. Clinically, cancer patients lose weight and appetite, which is associated with nausea and vomiting associated with chemotherapy [24, 25]. Based on this mouse model of IM, our experimental results showed that HPW could improve 5-FU-induced diarrhoea and gradually restore body weight and food intake without adverse effects.

In the present study, intraperitoneal administration of 5-FU caused significant structural damage to the small intestine. Villus atrophy inevitably affects intestinal absorption, which may be partly responsible for weight loss, as shown in Fig. 2A. HPW treatment significantly increased villus height and crypt depth in the jejunum and restored the morphology of villi and crypts, which may indicate that HPW promoted crypt cell regeneration and increased crypt cell migration to villi. Reconstruction of intestinal micromorphology promotes the recovery of absorption, which may lead to increased food intake, as shown in Fig. 2C. Other studies have indicated that the administration of Sijunzitang polysaccharides significantly altered the appearance and histopathological results during delayed healing of gastrointestinal ulcers, which is a process associated with polysaccharide-mediated promotion of crypt epithelial cell migration [26]. In addition, the water-soluble polysaccharides of rhubarb also protect intestinal mucosal cells from apoptosis by mediating antioxidant effects and maintaining the structure of villi and crypts [27]. Further investigations are therefore required to elucidate the protective mechanism by which HPW improves intestinal inflammation and mucosal damage.

Goblet cells, which are markers of the intestinal mucosal epithelium and secretory components of the intestinal mucosal barrier, are observed by PAS staining [28]. A reduced number of these cells represents damage to the mucosal layer of the small intestine, exposing the epithelial surface of the intestinal lumen to bacterial translocation [29]. Goblet cells and other small intestinal mucosal epithelial cells produce mucin, which covers the intestinal mucosal layer, is similar to the mucus gel that protects the gastrointestinal tract and is a vital component of the mucosal barrier [30]. SIgA, a major component of mucin, is the principal protective molecule of specific (acquired) immunity that is secreted to mucosal surfaces, which optimizes microbial groups, prevents them from adhering to mucosal surfaces, reduces toxin expression by intestinal pathogens and effectively prevents bacterial translocation [31]. Our data suggested that HPW could significantly upregulate the quantity of goblet cells and sIgA levels, suggesting that Wuguchong polysaccharide could alleviate small intestinal mucosal inflammation by enhancing mucosal barrier function.

We found histological evidence that HPW alleviates 5-Fu-induced IM by improving impaired intestinal barrier function and then provided biochemical evidence to support these results. DAO is an enzyme in intestinal epithelial cells that suppresses cell proliferation by reducing polyamine concentrations, whereas D-lac is a bacterial metabolite produced by the intestinal flora [32]. Basal levels of both factors are generally low in normal mammalian systemic circulation and are usually observed only in the gut. During intestinal infection and inflammation, intestinal wall permeability increases, the translocation of numerous microorganisms from the intestine to the circulation increases, and intraluminal DAO and D-lac easily enter peripheral blood through the intestinal mucosa [33]. Moreover, the intestine is the largest contact surface between the human body and the external environment and is the central organ associated with the stress response under stressful conditions [33]. Chemotherapy enterotoxic drugs can attack the gastrointestinal tract with excessive free radicals, leading to impaired metabolism in intestinal epithelial cells, damaged cell function and an inflammatory response [34]. Therefore, we also examined the oxidative stress-related indicator T-AOC. The results of our current study were consistent with previous findings that the serum levels of DAO and D-LAC in the 5-Fu-induced mucositis mouse model were higher than those in the control group, indicating that the intestinal permeability of mice was increased. The levels of T-AOC were also higher than those in the control group, suggesting an oxidative stress reaction. Moreover, HPW supplementation alleviated all three indicators, suggesting that HPW may alleviate IM by re-establishing intestinal barrier function and reducing the oxidative stress response.

Previously, we discussed the mechanism by which HPW alleviates IM from the aspects of the intestinal mucosal barrier and permeability. However, intestinal homeostasis is a dynamic process between internal and external environments, and the apical junctional complex plays a crucial role in maintaining intestinal homeostasis, similar to intestinal epithelial cells. These tight junction proteins, including the representative transmembrane proteins Claudin and Occludin, bind endothelial cells together by means of scaffold proteins and actin, forming the mechanical barrier of the intestinal tract [35]. Claudins prevent the unlimited flow of water and solutes, as well as the invasion of luminal antigens [36]. The interaction of claudin-1 with integrin in local adhesions is involved in regulating transport between cells and the extracellular matrix. Claudin-1 can regulate normal cell homeostasis under physiological conditions and promote the adhesion of migrated cells under pathological conditions [37, 38]. Occludin, which is the first tight junction protein discovered, is thought to regulate extracellular permeability by sealing adjacent cells [39]. In vitro studies reported that the restoration of high Occludin expression improved the molecular barrier function of pig intestinal epithelial cells (IPEC-J2 cells) [40]. A previous investigation confirmed that chemotherapy-induced intestinal barrier damage in IM mice as associated with decreased expression of occludin [41]. Our present study revealed decreased expression of Claudin-1 and Occludin in the intestines of mice treated with 5-FU, while the expression of these two tight junction proteins was restored in mice that were administered HPW. These results strengthen the relevant findings on the role of tight junction proteins in intestinal barrier function and confirm that HPW can promote the recovery of the intestinal mechanical barrier and ameliorate IM by regulating intestinal tight junction proteins. In addition, we investigated the expression of PCNA, which is involved in DNA replication and double DNA strand reconstruction. PCNA is considered a signature of cell cycle dynamics and proliferative activity [42]. Continuous proliferation of intestinal epithelial cells and subsequent enhancement of tissue recovery can attenuate intestinal inflammation [43]. Therefore, PCNA is a molecule for evaluating the intestinal epithelial barrier. In our current study, PCNA expression was reduced in the model group, and HPW administration upregulated this indicator. It was further confirmed that HPW could promote the recovery of the intestinal barrier.

A better understanding of the molecular mechanism that leads to IM could provide therapeutic methods for curing these drug side effects and boosting the absorption of the small intestine. In 2004, Sonis et al. proposed an overlapping five-step model to summarize the biological phases of mucositis: initiation, primary damage response, signal amplification, ulcer formation, and healing [44]. The formation of reactive oxygen species, inflammation, and apoptosis are the most important molecular events in the early stages of injury. Among these sequential molecular events, NF-κB has been considered to be one of the most important transcription factors associated with tumour toxicity and therapeutic resistance [45]. Activation of NF-κB can upregulate more than 200 different genes, and many of these genes may have mucosal toxicity [46]. TNF-α and COX-2, both of which are important target genes of NF-κB, are involved in the immune response to stress in the inflammatory cascade [47]. Thus, we preliminarily measured the expression of these three key molecules at the protein level. The data suggest that HPW can ameliorate the downregulated expression caused by 5-FU administration, which may be one of the molecular mechanisms by which HPW can improve inflammation in the small intestinal mucosa. In addition, other studies have demonstrated that TNF-α may initiate the infiltration of inflammatory cells into the intestine by decreasing the expression of occludin, leading to structural changes in tight junctions [48]. COX-2 destroys the collagen subepithelial matrix and epithelial basement membrane by activating matrix metalloproteinases, which further damages the small intestinal mucosal barrier [49]. Activation of NF-κB promotes the secretion of inflammtory cytokines. IL-1β is essential pro-inflammatory cytokines broadly involved in inflammatory processes. In contrast, anti-inflammatory cytokines IL-10 exert effect by a specific pathway in inflammation. Our experimental results showed that HPW could not only considerably decrease the levels of IL-1β but also increase IL-10 expression in IM mice.

For the past few years, an increasing number of studies have confirmed that the intestinal microenvironment maintains a dynamic balance between organisms and the microbiota. The intestinal microbiota contains multiple carbohydrate active enzymes (CAZymes), which participate in the metabolism of dietary polysaccharides and produce SCFAs that are beneficial to host health [50, 51]. Research shows that SCFAs (i.e., acetate, propionate, and butyrate) can be resorbed by intestinal epithelial cells and protect the intestinal barrier [52]. In addition, SCFAs can also improve 5-FU-induced small intestinal mucosal inflammation by improving the intestinal mucosal barrier and reducing the level of inflammation [53]. Our results are consistent with those of Flavia et al. [54], who found that 5-FU reduced SCFA levels in mouse faeces. After HPW administration, the levels of total SCFAs, acetic acid and butyric acid increased, indicating that microbial activity gradually recovered. Some studies have shown that Bacteroides can participate in the metabolism of polysaccharides to form succinic acid, which can be used as a single carbon source to produce acetic acid and other SCFAs [55]. In our previous study, it was found that supplementation with polysaccharides from Wuguchong could modify the obesity status of mice fed a high-fat and high-sugar diet by increasing the abundance of Bacteroidetes and decreasing that of Firmicutes [13]. Therefore, we hypothesized that the beneficial effects of HPW on Bacteroidetes in mice might be one of the reasons for the increase in SCFAs in the current study. Some intestinal probiotics can degrade xylose and glucose in polysaccharides to form propionic acid [56]. However, there was no significant change in propionic acid levels in our study. We hypothesize that this is related to the fact that HPW comes from insects and has low xylose levels (3.21%, Fig. 1C). Moreover, butyrate acts as a health-promoting SCFA, providing energy to epithelial cells, enhancing mucosal barrier function and reducing inflammation [57]. In the present study, HPW could improve butyrate levels, but there was no statistical significance. This outcome is probably related to the dose and time of our intervention, which is also the focus of our follow-up study. There is moderate evidence [58] also revealed that the production of butyric acid was related to the metabolism of galactose and glucuronic acid. In our study, the proportions of these two monosaccharides reached 21.55% and 5.90%, which were relatively high levels. Therefore, the content of butyric acid increases after HPW treatment.

## Conclusions

Our results suggest that HPW, which is a bioactive polysaccharide extracted from insects, has a palliative effect on IM caused by drug side effects. HPW can alleviate 5-FU-induced mechanical damage to the intestinal barrier in mice, improve the expression of tight junction proteins, and reduce the activation of inflammatory pathways. Furthermore, HPW can also promote the levels of SCFAs, which are metabolites of the intestinal flora, and regulate intestinal microecology. In future studies, we will further purify this natural product and try to examine the mechanism of action in vitro.

## Data Availability

The datasets used and/or analysed during the current study are available from the corresponding author upon reasonable request.

## Abbreviations

HPW: Homogeneous polysaccharides from Wuguchong
IM: Intestinal mucositis
PCNA: Proliferation cell nuclear antigen
D-LAC: D-lactate
DAO: Diamine oxidase
SCFA: Short-chain fatty acid
5-FU: 5-Fluorouracil
HE: Haematoxylin–eosin
PAS: Periodic acid-Schiff
T-AOC: Total antioxidant capacity
GC–MS: Gas chromatography–mass spectrometry
